# Physical activity counseling in medical school education: a systematic review

**DOI:** 10.3402/meo.v19.24325

**Published:** 2014-07-24

**Authors:** Marie L. Dacey, Mary A. Kennedy, Rani Polak, Edward M. Phillips

**Affiliations:** 1 Department of Psychology, School of Arts & Sciences, MCPHS University, Boston, MA, USA; 2 Institute of Lifestyle Medicine, Joslin Diabetes Center, Boston, MA, USA; 3 Department of Family Medicine, Maccabi Health Service, Jerusalem, Israel; 4 Department of Family Medicine, Hebrew University, Jerusalem, Israel; 5 Physical Medicine and Rehabilitation, Harvard Medical School, Boston, MA, USA

**Keywords:** medical school education, physical activity, exercise, counseling, systematic review

## Abstract

**Background:**

Despite a large evidence base to demonstrate the health benefits of regular physical activity (PA), few physicians incorporate PA counseling into office visits. Inadequate medical training has been cited as a cause for this. This review describes curricular components and assesses the effectiveness of programs that have reported outcomes of PA counseling education in medical schools.

**Methods:**

The authors systematically searched MEDLINE, EMBASE, PsychINFO, and ERIC databases for articles published in English from 2000 through 2012 that met PICOS inclusion criteria of medical school programs with PA counseling skill development and evaluation of outcomes. An initial search yielded 1944 citations, and 11 studies representing 10 unique programs met criteria for this review. These studies were described and analyzed for study quality. Strength of evidence for six measured outcomes shared by multiple studies was also evaluated, that is, students’ awareness of benefits of PA, change in students’ attitudes toward PA, change in personal PA behaviors, improvements in PA counseling knowledge and skills, self-efficacy to conduct PA counseling, and change in attitude toward PA counseling.

**Results:**

Considerable heterogeneity of teaching methods, duration, and placement within the curriculum was noted. Weak research designs limited an optimal evaluation of effectiveness, that is, few provided pre-/post-intervention assessments, and/or included control comparisons, or met criteria for intervention transparency and control for risk of bias. The programs with the most evidence of improvement indicated positive changes in students’ attitudes toward PA, their PA counseling knowledge and skills, and their self-efficacy to conduct PA counseling. These programs were most likely to follow previous recommendations to include experiential learning, theoretically based frameworks, and students’ personal PA behaviors.

**Conclusions:**

Current results provide some support for previous recommendations, and current initiatives are underway that build upon these. However, evidence of improvements in physician practices and patient outcomes is lacking. Recommendations include future directions for curriculum development and more rigorous research designs.

Physical activity (PA) is a cornerstone of health. There is a large evidence base to demonstrate a direct relationship between regular participation in PA and a markedly reduced risk of several chronic diseases, including heart disease, stroke, type 2 diabetes, and certain cancers (1). Furthermore, regular PA contributes to successful management of chronic diseases (2). Despite these known benefits, most people remain sedentary. In fact, the World Health Organization (WHO) has identified physical inactivity as the fourth leading risk factor for global mortality, causing an estimated 3.2 million deaths each year (3).

Physicians have been identified by multiple organizations as having an important role in potentially addressing the prevalence of inactivity and its impact on chronic disease. Healthy People 2020 has called for an ‘increase in the proportion of physician office visits that include counseling or education related to PA’ (4); the National PA Plan states that making ‘PA a “vital sign” for health care providers to assess and discuss with patients/clients’ is an immediate priority for the healthcare sector (5); and the American College of Sports Medicine (ACSM) has developed a global initiative, *Exercise is Medicine*, to encourage healthcare providers, [physicians] to ‘assess and review every patient's PA program at every visit’ (6). These recommendations are based on evidence that physicians, who are often viewed as credible and respected source of health-related information, can be powerful motivators to increase PA (7–10). Furthermore, those who are physically active themselves are more likely to counsel patients about exercise (11, 12) and as physicians see their patients regularly, averaging three visits per year, they are in a good position to provide continued preventive counseling and feedback (12).

Despite these findings and recommendations, PA counseling is still not uniformly included in office visits. Only 32% of patients in a 2010 survey indicated that they had received PA counseling at an office visit during the past year (13), and physicians note multiple barriers to conducting counseling, including insufficient knowledge and specific skills on how to effectively prescribe exercise (14). This lack of training is supported by several reports during the last decade of infrequent and inadequate medical education in PA counseling globally, both in developed (15) and developing (16) countries. A 2002 survey of US medical schools found that only 13% of 102 schools included PA and health in the curriculum, and only 6% had a core course or requirement related to exercise (17). Similarly, the US Institute of Medicine's 2004 statements on improving medical education noted that most medical schools did not effectively include PA in their curriculum (18). More recently, a 2012 review of 109 studies found that PA was the least addressed topic in health behavior counseling curricula for medical trainees in comparison to smoking, nutrition, alcohol, and drug use (19).

With the recognition that the prevalence of medical education in PA counseling was less than optimal, we sought to understand the character and quality of those programs that do include this in the curriculum. In Fall, 2012, one author (RP) conducted a preliminary search for relevant studies and literature reviews within medical and social science databases, including the online Cochrane Library database, using general key terms (e.g., PA, medical students). He also manually reviewed reference lists from previously published articles. The result of this preliminary search was that he found current systematic reviews of obesity education in medical schools (20, 21) and behavior change counseling programs for medical trainees at various levels (19). However, there were no published studies or systematic reviews specific to PA counseling educational programs for medical students. This preliminary literature search also reflected the dearth of rigorous experimental studies in medical education in general, which is often characterized by infrequent utilization of theory-driven, conceptual frameworks (22) and an overwhelming number of non-randomized study designs (22, 23).

Previous research, however, has also suggested certain curricular features that could increase the likelihood of improving PA counseling skills and eventually patient outcomes. For example, medical training that provides opportunities for students to develop and maintain regular PA behavior is likely to increase frequency of PA counseling in practice, as it has been found that, similar to practicing physicians, those students who are personally active are more likely to counsel (24). Second, the most effective general health behavior counseling curricula have included a combination of didactics and clinical practice experiences within either simulated or real clinical settings (19, 21). In addition, conceptual frameworks and counseling models that illuminate the complexity of behavior change have been incorporated into the most apparently successful programs (19–21). Theoretical models with the most support include the transtheoretical model (TTM), social learning theory, self-determination theory, and motivational interviewing (19, 20, 25, 26).

We decided to examine more closely how medical school programs have incorporated these three components (students’ personal PA behavior patterns, didactics with experiential practice experiences, and theory-based pedagogy) into PA counseling curricula. We also decided to assess the strength of study designs and the evidence for specific measured outcomes in reported experimental studies that aimed to increase PA counseling competencies.

## Methods

### Search strategy

In line with the Center for Reviews and Dissemination recommendations for undertaking reviews in healthcare (27) and similar guidelines specific to medical education (28, 29), we determined our inclusion criteria utilizing the PICOS (Population, Interventions, Comparators, Outcomes, Study design) format (Table 1). We limited our search to medical school programs, as a goal of this review is to inform curriculum development at this level of training. As our preliminary search suggested that there are recent innovations throughout the world, we conducted an international review, although we did limit it to studies published in English. Our PICOS criteria initially included studies with any measurable outcomes; however, as discussed below, we refined this later in our search to include only those studies with measurable outcomes that link directly to future PA counseling skills. Finally, as this is a rapidly evolving field of interest, we limited our search to those studies published since 2000.

**Table 1:** Inclusion and exclusion criteria for systematic review of the literature

Category	Inclusion criteria	Exclusion criteria
Population	Medical school students of any year from any medical school in the world	Medical trainees post medical school Qualified healthcare professionals Students of other healthcare professions (e.g., dieticians, nurses)
Interventions	Educational interventions that have components that address physical activity counseling, including (but not restricted to): exercise prescription knowledge providers’ personal exercise/behaviors counseling/coaching strategies for PA Could be in any academic-sponsored program that is delivered in any academic, clinical or community setting	Educational interventions that do not have a component that addresses physical activity counseling (e.g., understanding muscle movement, cardiac physiology)
	Use any kind of educational methodology including (but not restricted to): theoretical lessens role plays workshops Personal experience	
	Instructed by any teacher including, but not restricted to: physician physiotherapist physiology psychology social worker fitness guide	
Comparators	Studies with or without control/comparison groups	Not applicable
Outcome	Studies with at least one outcome measure that addresses future physical activity counseling.	Studies without any reported outcome data Studies with outcomes that do not link to future physical activity counseling
Study design (and study feature)	Any design (except studies without outcome measures) English language only Published 2000–2012	Insufficient detail to determine any study content

**Fig. 1:**
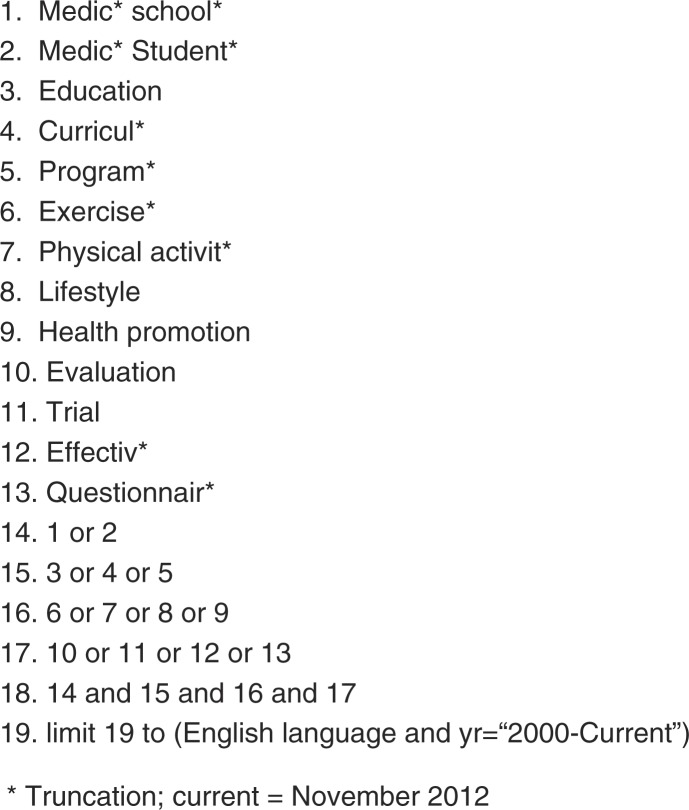
Search terms.

PICOS inclusion informed the selected search terms (Fig. 1). We defined these together with professional library staff. Terms are based on the target population (medical students), interventions (educational sessions addressing future PA counseling), and outcomes (program evaluations). We did not include terms related to comparators and study design because our preliminary search indicated that relevant studies would likely be excluded from the review if interventions lacked control or comparison groups. We combined search term sets (using AND) and exploded all search terms using the truncation (*). We also set language (English) and time frame limitations (publication date after January 1, 2000).

**Fig. 2:**
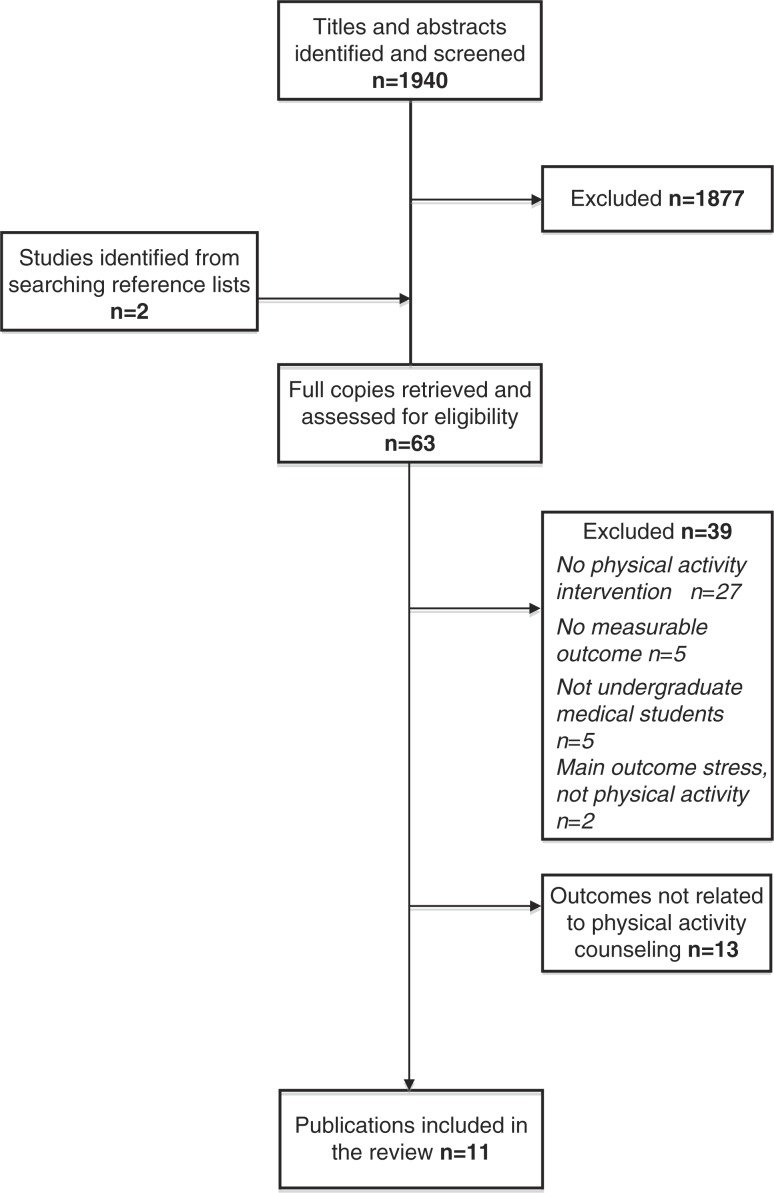
Study selection process for systematic review of physical activity counseling education in medical school.

Between November 2012 and February 2013, the first three authors (MD, RP, MK) systematically searched the MEDLINE, EMBASE, PsycINFO, and ERIC electronic databases for relevant studies using our search terms, and then eliminated duplicates. We imported the results of all searches to RefWorks^©^ and maintained relevant bibliographic databases using this software throughout the review process. Our initial findings (*N*=1,940) comprised results from these database searches as well as additional studies from reference lists of relevant articles (Fig. 2). The first three authors each independently screened the titles and abstracts of these articles, and excluded any that did not obviously meet our PICOS criteria. Studies with titles/abstracts that presented ambiguous information or did not indicate key details (e.g., identity of the study population) remained included so that relevant studies would not be missed. We also identified studies from reference lists (*N*=4). We compared our independent results and discussed inconsistencies to reach consensus of inclusion (*N*=63). During our final step, MD, RP, MK independently read these full reports to determine those that met PICOS inclusion (*N*=11). During this process, we also narrowed the outcomes criteria from PICOS to include only programs with evaluations that inform future PA counseling behaviors in order to adhere to our original objectives.

### Data extraction and synthesis

We conducted data extraction on each of the 11 identified studies, utilizing a tool developed by two researchers (MD, MK) that followed recommended guidelines (27). Two researchers (MD, RP) independently coded descriptive and methodological features of each study, which included study site, design, aim, participants, instructor professions, intervention components including setting and length, theoretical framework, measurement tools, and outcomes. They also conducted data extraction of PA components, including if and how each study addressed students’ personal PA behaviors and/or PA counseling. A third researcher (MK) independently reviewed coding and data extraction decisions. All three researchers collaboratively discussed and resolved decision inconsistencies.

### Program effectiveness: strength of study designs and measured outcomes

**Fig. 3:**
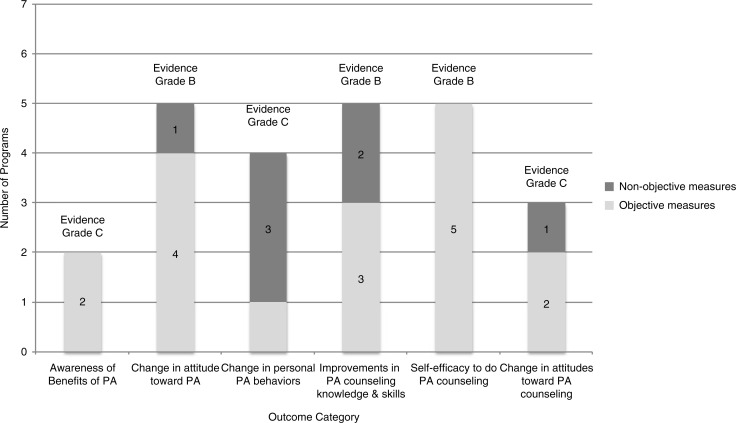
Number of programs using objective vs. non-objective measures reported by outcome.

In order to assess the overall effectiveness of the reviewed educational interventions, we first evaluated each study's design (Table 2), and then we assessed the strength of evidence for a number of measured outcomes shared by multiple studies (Table 3, Fig. 3). In regard to individual study design, we applied an approach utilized previously (20), and three researchers (MD, MK, RP) independently evaluated and reached consensus on intervention transparency and control for risk of bias in each study. Intervention transparency refers to whether the educational program and evaluation procedures were described sufficiently for replication. Control for risk of bias refers to whether a study included baseline measurements and/or a control or comparison group.

**Table 2:** Study design and quality of 10 programs included in systematic review

		Intervention transparency (description sufficient for replication)		Control for risk of bias	
		
Source	Study design	Program	Evaluation	Baseline measures	Comparison group
Barss et al. (34)	Post only survey		✓		
Bass et al. (35)	Before-and-after test	✓	✓	✓	
Conroy et al. (36)	Before-and-after survey			✓	✓
Frank et al. (33)	Retrospective qualitative (focus groups)	✓	✓		
Frank et al. (32)	Interrupted time series survey (baseline, mid, end of program)	✓	✓	✓	✓
Grant et al. (37)	Post only semi-structured family interviews				
Kushner et al. (38)	Post only questionnaire	✓	✓		
Mohler et al. (39)	Post only survey	✓	✓		
Moser et al. (40)	Post only survey	✓	✓		
Pandejpong et al. (41)	Post only medical chart comparisons and surveys				✓
Wagenschutz et al. (42)	Post only survey including retrospective data	✓	✓		

**Table 3:** Evidence of effectiveness for six measured outcomes in systematic review

Measured outcomes	Overall evidence rating	Quality	Quantity	Consistency
PA				
Awareness of benefits of PA	C	C	C	A
Change in attitude toward PA	B	B	A	A
Change in personal PA behaviors	C	C	A	A
PA counseling				
Improvements in PA counseling knowledge & skills	B	B	A	A
Self-efficacy to do PA counseling	B	B	A	A
Change in attitudes toward PA counseling	C	C	B	B

To determine the collective strength of evidence, we adapted a numerical scale used previously to evaluate educational outcomes common to several studies (30). This scale incorporates guidelines to rate the strength of scientific evidence (31) and recommendations on how to apply these to educational interventions (23). Using this approach, we first determined the primary measured outcomes related to PA and PA counseling skills investigated by the studies under review. Then, taking all studies into consideration, we objectively graded the quality, quantity, and consistency of evidence for each measured outcome. Our grading protocol was developed and piloted by two researchers (MD, MK), and then independently completed by three researchers (MD, MK, RP). Consensus was reached after resolving one discrepancy in scoring.

For grading the quality of evidence for each primary measured outcome, two factors were evaluated: utilization of control or comparison groups and the presence of objective assessments (30). Objective assessments are those that were quantifiable. We determined that objective assessments could include self-report Likert scales or other quantifiable survey data for measuring personal reports of cognitive change, such as self-efficacy, awareness, and attitudes. However, more observable outcomes, such as knowledge, skills, and behaviors, required other tools (e.g., skill tests, observer checklists, adherence to guidelines) to be considered objective assessments. Results from focus groups were considered as qualitative and not objective. Also, self-reports of general improvements in knowledge, skills, or behaviors were deemed non-objective assessments.

As stated above, we applied an evidence grading system utilized previously (30). For quality of evidence to be graded A, there must be at least one study utilizing a randomized controlled trial AND at least 75% of the studies that assessed the outcome of interest used objective assessment methods. To meet the criteria for grade B, there must be at least one study that measured the outcome utilizing a control or comparison group (not necessarily randomized) AND at least 50% of the studies used objective assessments. For grade C, there is at least one study with a control or comparison group OR at least 50% of the studies used objective assessments. Quality grade of D was assigned when neither criteria for grade C was met.

Grading for quantity of studies for each primary measured outcome was determined by the number of studies investigating each outcome. Grade A was conferred when at least four studies measured the outcome; grade B was assigned if there were three studies; grade C was applied if there were two studies.

Grading for consistency of results for each primary measured outcome was determined by the percent of studies that reported findings consistently in the same direction (beneficial, no change, or harmful). Grade A indicated that at least 75% of studies show an effect in the same direction; grade B was assigned if there were 51–74%; grade C indicated 25–50%.

The overall evidence rating for each measured outcome is the lowest grade for any of the three criteria factors of quality, quantity, and consistency (Table 3).

## Results

Eleven study reports represent 10 educational programs, as one program conducted two studies to investigate separate outcomes (32, 33). Frank, Smith, and Fitzmaurice (33) discuss the curriculum overview and qualitative findings, while Frank, Elon, and Hertzberg (32) provide quantitative results for the same program. Characteristics of each of these 10 programs are provided in Table 4.

**Table 4:** Program characteristics of 10 medical school educational interventions that address physical activity counseling skills included in a review of the literature

Source	Program duration	Educational intervention	No. of respondents/eligible study participants	Measured outcomes and evaluation tools related to PA counseling	Results
Barss et al. (34)	6 months	Lifestyle curriculum addresses main national determinants of health, disease, and injury through five lectures, and home-based observational interviews with families re: family's safety, smoking, food hygiene, and health behaviors. Students also prepare and deliver oral presentations on a self-selected lifestyle topic. Assess personal lifestyle using 1 week activity and nutritional log.	43/50	Survey assessment of self-reported changes in awareness of benefit of exercise and improvement of counseling skills Survey assessment of self-reported changes in personal PA behaviors Survey assessment of curriculum value	Improved awareness of benefit of exercise: 96% strongly agree/agree; Improved PA counseling skills: 100% strongly agree/agree Increased personal PA behavior: 46% started to exercise regularly; 63% started using stairs more frequently Curriculum-improved skills and stimulated interest in Community Medicine: 84% strongly agree/agree
Bass et al. (35)	2.5 hours	Nutrition and health promotion unit includes 2 hour interactive lecture with videos and case scenarios on PA and nutrition counseling and two 15 min simulated patient encounters to practice PA and nutrition counseling skills.	57/115	Assessment of changes in knowledge, self-reported confidence for PA counseling, and attitude toward utility and necessity of PA counseling	Significant improvements on case-based knowledge test, and 10-point Likert confidence scale (*p*<0.001) No significant change in attitude re: utility and necessity of PA counseling
Conroy et al. (36)	14 weeks/28 hours	Preventive medicine and nutrition course consists of weekly 45 min lectures and 90 min small group problem-based tutorials with simulated cases to teach counseling skills. One week specific to exercise. Assess personal PA and nutrition	110/137	Survey assessment of changes in self-reported personal PA behaviors and self-efficacy for PA counseling	18% reported course had changed exercise habits Significant improvement in self-efficacy for PA counseling on 4-point Likert scale ( *p*<0.001)
Frank et al. (33)	4 years	‘Healthy-doc Healthy-patient’ project addresses disease prevention through multiple lectures and activities incorporated into existing curriculum, including personal health practices and standardized patient counseling. Optional extracurricular programs and activities support personal health practices.	15/75 (Qualitative)	Focus groups’ input on changes in habits and attitudes related to personal health behaviors Focus groups’ input on program's impact on clinical prevention practice Focus groups’ input on curriculum quality/effectiveness	Overall positive feedback on program's influence on personal health habits and attitudes, despite limited recall of lecture about exercise 2 years later and negative feedback on program intensity. No pattern on opinions regarding utility of program in developing clinical practice skills Least favorable perceptions and recall of lectures; more positive regard for and recall of non-lecture components integrated into existing curriculum; most positive about extracurricular, student-initiated optional activities.
Frank et al. (32)		Same program as Frank et al. (33)	106/114 (Quantitative)	Pre-, mid-, post survey assessment of changes in self-reported compliance to CDC PA recommendations Post only faculty rating of standardized patient counseling, i.e., none, minimal, more extensive	Non-significant decrease in compliance to CDC PA recommendations mid- to post-intervention: control (64–50%), intervention (71–66%) Significant increase in frequency of exercise counseling during standardized patient counseling (*p*=0.03); 50% more likely to counsel than control
Grant et al. (37)	9 months	Family Studies Program promotes students’ understanding of family functions and its role in health promotion and students’ communication skills through pre-placement background reading and workshops, followed by 10–12 student visits to assigned family to learn about families’ day-to-day functions and assist in development of self-management practices.	30/30	Nurse administered semi-structured questionnaire with families to assess their self-reported changes in exercise habits in response to program	100% of families said knowledge of personal health issues had increased 66% of families reported positive changes in lifestyle behaviors 66% of families reported better understanding of importance of maintaining ideal weight
Kushner et al. (38)	6 weeks/12 hours	Behavior Change Project (BCP) within Healthy Living unit promotes students’ well-being and self-care through 6 weekly 2-hour didactic/experiential sessions on behavior change principles Students set personal goal and monitor progress in changing a self-selected health behavior	343/343	Survey assessment of which behavior students chose to change and goal achievement success rate. Survey assessment of attitude toward project's utility and its burden on students	44% selected exercise among six possible health behaviors. 39% achieved exercise goal Utility of project: Average overall student score 4.0/5 on Likert scale, e.g., project was valuable, learned to be healthier. Burden of project: Average overall student score 2.8/ 5 on Likert scale, e.g., monitoring behavior was difficult and time consuming
Mohler et al. (39)	2 hours	Healthy Aging Rounds promotes students’ knowledge of exercise prescription, importance of social engagement, and counseling skills with older adults. Rounds include background readings and two 1-hour didactic/experiential sessions that include 20-min student/healthy older adult mentor practice counseling.	26/37	Survey assessment of student and mentor opinion of program quality Survey assessment of students’ change in attitude toward role of PA in aging, and importance of exercise prescription for older adults	Quality of program Good/excellent: 92% of students and 97% of mentors 85% moderately or much more positive attitude regarding role of PA and importance of exercise prescription with older adults
Moser et al. (40)	4 week/60 hours	Health Beliefs and Behavior course promotes understanding of health behaviors and provides counseling tools through classroom didactics, workshops, and experiential learning in clinical ambulatory setting. Students participate in self-selected behavior change exercise.	149/150	Survey assessment of self-reported changes in attitudes, knowledge, and counseling skills	Improvement in open-mindedness toward behavior change, understanding of health behavior principles, and ability to recommend behavior change strategies:: Average overall student score 4.2/5 on Likert scale
Pandejpong et al. (41)	3 months/3 sessions	Continuity of Care Clinic (CCC) to improve longitudinal cardiovascular risk management skills during Internal Medicine rotation includes patient encounters and follow-up visits under supervision of attending physicians.	38/192 (for chart review) 192/192 (CCC satisfaction survey) 22/? (physician supervisor survey)	Three month post-graduation medical chart audits for compliance to standard quality of care for Hypertension, Diabetes and Dyslipidemia using 12-task checklist compared performance between CCC and non-CCC program graduates Three month post-graduation survey assessment of students’ self-report of changes in confidence, understanding, and attitude toward cardiovascular risk management, and possible adaptation to personal practice Three month post-graduation survey assessment of physician supervisors comments compared CCC and non-CCC graduates’ confidence, competence, and attitude re: cardiovascular risk management	Significant difference between CCC and non-CCC graduates in ‘recommending lifestyle modifications’ (*p*<0.001) Strongly agree/agree improved confidence (86.8%), understanding (93.7%), and attitude (84.6%) toward cardiovascular risk management, and could adapt knowledge to personal practice (83.6%) on 5-point Likert scale Strongly agree/agree CCC graduates more confident (64%), competent in patient management (64%), competent in longitudinal care (51%), and have better attitude toward cardiovascular risk management (72%) compared to non-CCC graduates on 5-point Likert scale.
Wagenschutz et al. (42)	1 week	Two health behavior counseling interaction programs promote tobacco cessation (TCC) and nutrition/PA (NPA) counseling skills through background reading and online interactive professional skill builder program followed by 2 (TCC & NPA) 1-hour simulated counseling sessions with feedback.	199/~340	Survey of self-reported changes in students’ comfort level (self-efficacy) in performing nutrition & physical activity counseling	Significant increase in comfort level in performing nutrition and PA counseling (3.79 retrospectively before intervention vs. 4.48 post-intervention on 5-point Likert scale, *p*<0.001)

### Curriculum structure and timing

Seven programs (70%) are from US medical schools. The remaining three (30%) are reports from United Arab Emirates (34), Bahrain (37), and Thailand (41). All projects are based at a single institution. Three programs (30%) were added to the existing curriculum as separate courses or programs (36, 37, 39), while seven (70%) were incorporated into already established courses and/or internships (32–35, 41, 38, 40, 42).

The programs vary in placement and length within the medical school curriculum. Four programs (40%) occur during the first 2 years (34–36, 38); five (50%) are during the last 2 years of medical school (37, 39, 41, 40, 42); and one program (10%) is integrated into all 4 years of medical school (32, 33). There is also considerable heterogeneity in the length of the programs, which ranges from 2 hours (39) to 4 years (32, 33).

### Curriculum design

#### Theoretical frameworks

Only five programs (32, 33, 35, 38, 40, 42) (50%) report that theories and counseling models, based in behavioral science research, inform curricula design. The most comprehensive report of application of health models is by Moser (40). In this interdepartmental 60-hour course during the third year, students learn constructs from the TTM, Health Belief Model, and Social Cognitive Theory, and they apply these with well-grounded strategies such as motivational interviewing and determination of stage-of-readiness for change. Wagenschutz (42), which includes background reading and two simulated practice sessions, also incorporates motivational interviewing, and the 5 A's (ask, advise, assess, assist, and arrange). The other three programs describe utilizing just one framework (35) in a short two and a half hour health promotion unit, and provide students with a description of TTM and an interview template for a simulated role-play activity, which includes determining stage-of-readiness. Frank's program (32, 33) notes the importance and inclusion of modeling, based on Social Learning theory, and Kushner (38) states that their behavior change plan project is grounded in behavioral theories that promote techniques such as goal setting, self-monitoring, and reinforcement.

#### Physical activity content and personal PA behavior

All educational programs (100%) include didactics and/or experiences with PA as a health behavior. PA is always incorporated into curriculum that includes other components such as population health (34), nutrition (35, 36, 42), health behavior change and counseling (38, 40), healthy aging (39), and/or disease prevention and management (32, 33, 37, 41). Five programs (50%) (32, 33, 37, 38, 41, 40) do not provide enough information in describing their curriculum to estimate the proportion of time dedicated specifically to PA. Among the other five programs, the proportion of time dedicated to PA appears to range from less than 10% (34, 36) to approximately 50% (39, 35, 42).

Five programs (50%) (32–34, 36, 38, 40) include opportunities for students to chronicle their own PA during the course, but it is not required in all these programs. Three programs (30%) (32, 33, 38, 40) require students to develop and implement health behavior change plans, but only Frank (32, 33) require PA as the health behavior. In others, they have the choice of self-selecting PA as an option among several health behaviors (38, 40). The length of time that students address their personal PA behaviors is highly varied, ranging from 1 week (34) to over 1 year (32, 33); the sole longitudinal project also includes extracurricular exercise-related events, for example, yoga and walk/runs.

#### PA counseling skills and experiential learning

All programs (100%) include both didactics and experiential approaches; nine of these (90%) include opportunities for PA counseling practice. The other program (38) addresses behavior change principles and personal health behaviors but not counseling skills per se. Counseling practice in these nine programs varies in setting and type. Five programs (50%) (34–37, 39–41) include practice with patients/clients, which takes place in either community homes (34, 37), medical (40, 41), or classroom (39) settings. Five programs (50%) give students counseling experience without patient/client contact, but rather these employ role-playing (32, 33, 35, 40, 42), or problem-based tutorials with simulated cases (36). Three programs (30%) (35, 40, 42) specifically note the importance of patient-centered counseling skills.

### Strength of study designs

All study designs (Table 2) are quasi-experimental in that participants are not randomized to intervention or control groups; this approach is common in educational research (23). In terms of intervention transparency, six programs (60%) provide description of both the curriculum and evaluation procedures, which would allow replication (32, 33, 35, 39–42). Control for risk of bias is limited. Only three studies (30%) provide baseline measures with pre/post differences (32, 35, 36), while the remainder (70%) employ a post-intervention only evaluation. Three studies provide both qualitative and quantitative results (32, 33, 37, 38). Only the educational intervention designed and implemented by Frank (32, 33) meets all criteria related to intervention transparency and control for risk of bias. Also, the longitudinal nature of this program, multiple assessment points, and the combination of quantitative and qualitative outcomes all further contribute to its relative strength.

Three studies (30%) utilize naturally occurring cohort controls, that is, other medical student groups, as comparison samples (32, 36, 41), and these study designs all present some barriers to optimal intervention-control comparisons. Two of these three programs include baseline measurements (32, 36). Conroy (36) collected data from medical students in the same year (second) as the intervention group, who were enrolled in a different program with a separate preclinical curriculum. While the two groups are matched well with no significant baseline differences in demographics or any outcome measures, the intervention group size is much larger than the comparison group (*N*=134 vs. 23 at baseline; *N*=110 vs. 13 for pre–post linked data). Frank's (32) quantitative analysis has a comparison group from the same program, which was 1 year further advanced. While the groups are similar in most baseline demographic characteristics, they differ in several opinion and attitude measures, including the intervention group's greater interest in prevention-oriented specialties. Finally, Pandejpong (41) compares outcomes for physicians who had participated in the continuity of care clinic curriculum (CCC) while in medical school with physicians who did not. As this study involves assessment and comparison of patients’ medical charts after students graduated, there are no data available on the demographics or other baseline characteristics of the non-CCC comparison group, so the homogeneity of these two groups is uncertain.

### Strength of evidence for measured outcomes

We determined that six measured outcomes were most often evaluated and relevant to our review question (Fig. 3 and Table 3). These are students’ awareness of benefits of PA, change in students’ attitudes toward PA, change in personal PA behaviors, improvements in PA counseling knowledge and skills, self-efficacy to conduct PA counseling, and change in attitude toward PA counseling.

An increase in students’ awareness of benefits of PA received an overall C rating. Only two programs (34, 40) assessed this outcome and neither study utilized a control group or collected baseline data, so control for risk of bias was low. Yet both studies used acceptable objective measures for this kind of outcome, that is, quantified survey data, and students consistently reported improvements in their awareness of the benefits of PA.

Change in students’ attitudes toward PA received an overall B rating. Five programs (33, 38–41) assessed this outcome, and four (38–41) of these five studies provided objective, quantified survey data. One study (41) also had external observers rate students in comparison to controls not exposed to the educational program. While Frank (33) provided input from focus groups, thus not objective, the comments of these students generally supported the consistent positive reports of attitude change found in all the other studies. No studies had baseline data, as this outcome was assessed solely at the end of programs.

Change in personal PA behaviors received an overall C rating. While there are four programs (32–34, 36, 38) that measure this outcome, only one study (32) uses an objective assessment strategy, that is, reported compliance to CDC PA guidelines. It is notable that data collected by Frank (32) indicate a non-significant decrease in students’ compliance to CDC PA guidelines between the midpoint and end of this 4-year study, which counters focus group reports from Frank (33) suggesting that personal exercise habits had improved. All other programs consistently report some increase in personal PA behaviors, and two had pre/post comparisons (32, 36).

Improvements in PA counseling knowledge and skills received an overall B rating. Five programs measure this outcome (32–35, 40, 41), and they consistently indicate that students have improved in this area. Three programs use objective assessments (32, 35, 41), that is, accuracy of recommendations for case studies (32, 35, 41), and standardized faculty ratings for role play (32) and clinic-based (41) patient encounters. Two programs (32, 41) also utilize comparison groups as controls, which contribute to the strength of this finding.

Self-efficacy to do PA counseling also received an overall rating of B. Five programs evaluate this outcome (35, 36, 40–42) utilizing various Likert scales, and all consistently demonstrate improvements in this area. Two programs with baseline data (35, 36) support this finding by demonstrating statistically significant pre/post changes. Also, two programs that include comparison groups as controls (36, 41) demonstrated that improvements in the intervention group were greater than any changes in the comparison group. Overall, these findings support improvements in students’ self-efficacy to conduct PA counseling as a result of these educational interventions.

Changes in attitude toward PA counseling received an overall rating of C. Three programs assess this outcome (33, 35, 39). None of these studies include controls for this outcome, and only two utilize objective measures to assess it (35, 39). Two programs (33, 35) indicate that students’ attitudes had not changed, while survey results from the third study (39) show that 85% of students indicated a more positive attitude toward PA counseling. Thus, findings for this outcome are the most inconsistent of all the measured outcomes in this review.

### Program feedback

Student opinion regarding program quality was also measured in five programs (50%) (32–34, 38, 39, 41). All but one program (41) asked for feedback immediately after completion of the program. Students were also surveyed during the program (32) and 3 months post-graduation while in clinical practice (41). Students’ self-reports of changes in attitude toward PA and increased confidence and understanding around counseling were consistently positive. Students reported that they consider this topic as worthwhile and important to their medical education. Faculty (41), mentors (39), or supervisors (41) also provided feedback on student skills. These observers overall reported improved counseling skills in those students who participated in these programs.

## Discussion

Our review of medical education literature on the impact of PA counseling curricula published 2000–2012 resulted in locating 10 programs in four different countries with reports on outcomes. One program provided two evaluation study reports (32, 33). These programs had considerable heterogeneity, and study interventions and outcomes were not always sufficiently described to support replication and optimal assessment of program quality and effectiveness. Similar design limitations have been reported in systematic reviews of obesity and behavioral counseling education (19–21) and noted as common weaknesses of medical education intervention studies (22, 23). We accommodated these limitations by utilizing recommended approaches to quantify available information as much as possible (20) and to evaluate the collective impact on a select group of measured outcomes (23, 31). We also specifically examined to what extent these programs have complied with previous recommendations to address personal PA behaviors, utilize conceptual models, and provide both didactics and experiential learning opportunities.

We identified four programs that show strong overall evidence (B level) of improvements in students’ attitudes toward PA in general, their PA counseling knowledge and skills, and their self-efficacy to conduct PA counseling (32, 33, 35, 40, 41). In reviewing the elements of these four programs, we found that program duration ranges from 2.5 hours (35) to 4 years (32, 33), indicating that programs of varying length and intensity may have impact at least in terms of immediate outcomes. These four programs also generally follow previous curriculum recommendations. All include opportunities to practice PA counseling, either in simulated encounters (32, 33, 35) or clinical settings (40, 41); three programs (32, 33, 35, 40) have interventions that are grounded in conceptual frameworks, and two programs (32, 33, 40) provide opportunities for students to address their own PA behaviors. Furthermore, these four programs with the strongest evidence for impact all integrate PA into existing programs that address behavior change or an array of health promotion and disease prevention topics. Thus, there is some support for developing PA counseling skills by blending this topic into a range of types of existing curricula.

Recent initiatives to expand and improve medical education in PA counseling are demonstrating adherence to the most supported findings from our review. The ‘Exercise is Medicine’ (6) Education Committee of the American College of Sports Medicine, chaired by one author here (EP), has formally recommended knowledge, skills, and abilities (KSAs) related to PA that all medical students should accomplish. These include two competencies highlighted by our review, that is, students’ personal PA behaviors and counseling strategies based on conceptual frameworks (43). One medical school in the United States is incorporating these KSAs as the foundation of a new exercise-based curriculum (44), while also integrating experiential patient-centered activities into the existing curriculum. Further, key stakeholders and institutions are striving to move PA counseling curriculum forward within the United States. A 2013 national Lifestyle Medicine Think Tank (45) recently examined how to best integrate the KSA competencies into US medical school curricula. They followed this with a presentation of vision, goals, and strategies at a Bipartisan Policy Center public meeting on teaching nutrition and physical in medical school (46). These recent efforts to advance medical education in PA counseling overall are well-grounded in the available evidence.

However, as stated above, the educational intervention studies within this review generally have design limitations, which put constraints on our ability to determine the full extent of impact on students, their clinical skills, and especially their future patients’ health. There were no randomized, controlled trials; rather they are all quasi-experimental designs. Only three of the 10 programs reviewed have naturally occurring comparison groups (32, 33, 36, 41), which are equivocally matched and assessed. Also, generalizability is limited. All programs occur within single institutions rather than multi-institutions, and there is considerable heterogeneity in program content, structure, and intensity, that is, program duration and frequency of exposure to PA. Finally, clinical outcomes are measured for only one intervention (41), and this is physician performance post-graduation, not patient behaviors. Thus, how these educational programs eventually translate to actual clinical professional practice, resultant patient behaviors, and improved health outcomes still remains largely unknown.

We recommend that curriculum development in PA counseling medical education utilize the most strongly supported findings, as noted above (and as recently enacted through KSAs and related initiatives). Concurrently, however, we recommend that the current limitations in medical education research be recognized and that curricular developers seek to establish a stronger evidence base for educational programming. Specifically, educators should use controlled designs in which new programs at least do pre/post comparisons of student groups matched as closely as possible to the intervention group. We also support the development, pilot testing, and consistent use of a set of valid and reliable tools across programs to measure impact, which would offset the array of researcher-generated tools characteristic of the studies we reviewed. Also, measures should be objective and observable wherever possible. Most of the current programs relied on self-report and survey Likert-type measures. While these are suitable for assessing attitude changes, including self-efficacy, more objective evaluations could be used to measure actual changes in behavior, knowledge, and skills (e.g., case study tests, supervisor checklists, daily logs on phone apps, compliance to federal recommendations). Finally, these evaluations should extend to clinical professional practice, patient behaviors, and health outcomes in order to establish the strongest evidence base for medical training in PA counseling.

We acknowledge that there are limitations in this systematic review. Primarily, given the considerable heterogeneity in the reviewed studies, and the fact that no more than five programs studied any specific measured outcome, our evaluation of educational effectiveness was inherently limited. Further, as study authors did all coding, the subjective nature of this includes some potential for coder bias despite independent evaluations. Also, we used criteria to evaluate study designs and grade evidence of strength of studies that were not specifically developed to assess medical education programs. Rather, we adopted approaches from previous healthcare education reviews (19, 20, 30), which typically were based on criteria for evaluating clinical interventions. Also, only published studies were reviewed so there could be bias toward favorable results. Any number of medical educational programs might exist that address PA counseling, which have not been evaluated and disseminated. Finally, the exclusions in our search criteria potentially could have eliminated relevant studies.

In conclusion, the available evidence supports the potential effectiveness of the inclusion of PA counseling in medical school education. Our recommendations for curriculum development based on this review, previous research, and current initiatives are to 1) address students’ personal PA behavior patterns; 2) incorporate strong conceptual base into counseling curriculum; 3) provide both didactics and counseling practice experiences; 4) integrate PA training into other topics and the existing medical school curriculum; 5) support opportunities for shorter as well as more intense programs; and 6) develop multi-institution programming. This review also highlights the need for more rigorous research in this curricular area. Thus, PA counseling program evaluations should follow previous recommendations for evidence-based research designs in medical education (22). Ongoing advancements in the design and evaluation of PA counseling education in medical schools will no doubt result in more effective physician practices in lowering risk and improving management of chronic diseases.
